# Non-signalling energy use in the developing rat brain

**DOI:** 10.1177/0271678X16648710

**Published:** 2016-07-20

**Authors:** Elisabeth Engl, Renaud Jolivet, Catherine N Hall, David Attwell

**Affiliations:** 1 Department of Neuroscience, Physiology & Pharmacology, University College London, London, UK; 2 CERN, and Département de physique nucléaire et corpusculaire (DPNC), University of Geneva, Geneva, Switzerland; 3 School of Psychology, University of Sussex, Falmer, UK

**Keywords:** ATP, brain development, brain slice, energy metabolism, lipids

## Abstract

Energy use in the brain constrains its information processing power, but only about half the brain's energy consumption is directly related to information processing. Evidence for which non-signalling processes consume the rest of the brain's energy has been scarce. For the first time, we investigated the energy use of the brain's main non-signalling tasks with a single method. After blocking each non-signalling process, we measured oxygen level changes in juvenile rat brain slices with an oxygen-sensing microelectrode and calculated changes in oxygen consumption throughout the slice using a modified diffusion equation. We found that the turnover of the actin and microtubule cytoskeleton, followed by lipid synthesis, are significant energy drains, contributing 25%, 22% and 18%, respectively, to the rate of oxygen consumption. In contrast, protein synthesis is energetically inexpensive. We assess how these estimates of energy expenditure relate to brain energy use in vivo, and how they might differ in the mature brain.

## Introduction

Energy availability limits information processing in the brain,^1–3^ which consumes energy disproportionately in relation to its fraction of total body mass.^4,5^ The brain's most salient feature is neuronal communication, and the energetic cost of the different cellular processes underlying signalling has been well described.^1–3,6–11^ Most energy use is on the removal of sodium ions that enter neurons to generate synaptic and action potentials.^1,6,11^ However, inhibiting the sodium–potassium ATPase, without which signalling activity ceases, has shown that around 45% of the brain's baseline energy use in vivo is consumed on non-signalling processes,^12^ which is considerably more than is estimated or assumed in many models of brain energy use.^1,11,13,14^ Surprisingly, it is largely unknown which non-signalling processes consume the rest of the brain's energy.

Often called ‘housekeeping’ processes, the many tasks that the brain performs in addition to signalling provide the scaffold on which signalling, plasticity and the encoding of memory can occur. The actin cytoskeleton regulates the morphology of the mature neuron, as well as its growth in development,^15–18^ and modulates synaptic function.^19,20^ To do this, the actin cytoskeleton treadmills continuously, by adding G-actin monomers to one end of strands of F-actin and releasing them at the other end.^21^ Along the F-actin polymer, ATP bound to G-actin gets hydrolysed to ADP.^22^ Bernstein and Bamburg^23^ claimed that actin cycling accounted for half of all energy use in neuronal cultures but, as their experimental method suppresses neuronal glutamate release, this fraction is likely to be overestimated.^24^ Conversely, modelling of actin and microtubule turnover suggested that less than 1% of total brain energy use was spent on actin treadmilling, and even less on microtubule turnover.^24^ Experimental data on the energetic cost of microtubule turnover are lacking, even though microtubules also exist in a state of dynamic instability, growing and shrinking in an energy-dependent manner, and hydrolysing tubulin-bound GTP along the way.^25–28^

Alongside continuous actin and microtubule restructuring, phospholipids and proteins must be synthesised in the brain. Experimental data for the energy use of either process are scarce, but protein synthesis was estimated theoretically to account for no more than 2% of the total consumption of ATP in the brain^1,29^ and retina.^14^ While phospholipid synthesis was once thought to be similarly inexpensive,^14,30^ Purdon and Rapoport^31^ calculated that up to 25% of total brain ATP may be used on phospholipid metabolism, mainly on maintenance of the phosphorylation state of lipids (12%), with another 8% of total ATP spent on transport of phospholipids through the phospholipid bilayer to maintain lipid asymmetry across the membrane,^32^ and 5% on recycling and incorporation of short-lived fatty acids inside phospholipids.

Here, we present the first experimental evidence investigating, with a single approach, the relative contributions of the main non-signalling processes to the developing brain's energy budget. We blocked actin treadmilling, microtubule turnover, or lipid or protein synthesis in developing rat brain slices. By measuring oxygen level changes with an oxygen-sensing microelectrode as a proxy for energy expenditure, and modelling oxygen use throughout the slice, we could estimate the energy used on each process.

## Materials and methods

### Slice preparation

Experimental protocols were approved by UCL's Animal Welfare and Ethical Review Body. Procedures complied with the regulations of the Animals (scientific procedures) Act 1986 and reporting follows the ARRIVE (animal research: reporting of in vivo experiments) guidelines. Following cervical dislocation (no anaesthesia was required for animals of the age and size used), hippocampal slices (300 µm thick) from male and female P10 rats were cut on a Leica VT1200 S vibratome using ice-cold slicing solution containing (mM) 124 NaCl, 10 D-glucose, 26 NaHCO_3_, 2.5 KCl, 1 NaH_2_PO_4_, 2 MgCl_2_, 2.5 CaCl_2_ and 1 kynurenic acid (bubbled with 95% O_2_/5% CO_2_). The dissection followed the ‘magic cut’ method to maintain synaptic connectivity, using an angle of 10° for the ‘magic cut’ to preserve the CA1 region.^33^ Slices were used one to five hours after slicing. HEPES-buffered artificial cerebrospinal fluid (aCSF), to which blockers were added, was bath-applied (at 2.5 ml/min), while submerged slices rested on the glass bottom of a bath on the Zeiss LSM 710 microscope stage. The bath volume was 2 ml, and solution flowed over the top, but not the bottom, of the slice. Slices and electrode placement were observed using a ×25 lens. The aCSF pH was adjusted to 7.4 with NaOH, and contained (mM) 140 NaCl, 10 D-glucose, 2.5 KCl, 10 HEPES, 1 NaH_2_PO_4_, 1 MgCl_2_ and 2 CaCl_2_ (osmolarity 300 mmol/kg, bubbled with 100% oxygen, and heated to 36–37℃).

### Pharmacology

To establish whether spontaneous signalling had a detectable effect on O_2_ consumption, postsynaptic currents were inhibited with 10 µM NBQX + 50 µM D-AP5, presynaptic transmitter release and postsynaptic events were blocked with 250 µM cadmium, or action potentials and evoked synaptic events were inhibited with 1 µM TTX.

Actin polymerisation was reversibly blocked with cytochalasin D, which binds to the plus end of F-actin and prevents G-actin monomer attachment.^34^ Cytochalasin does not affect action potential propagation and duration in cardiac muscle cells.^35^ Cytochalasin D was dissolved in DMSO and made up in aCSF to a final concentration of 10 µM. When cytochalasin is applied extracellularly, maximal inhibitory effects are achieved with a concentration of 10 µM, and the time needed for maximal efficacy is three to four minutes.^34^ In order to verify that cytochalasin D does indeed inhibit actin treadmilling, we imaged isolectin B_4_ labelled microglia in hippocampal slices (see Supplementary Methods and Supplementary Figure 1). At rest, microglia constantly survey the brain by moving their processes.^36^ If actin treadmilling is inhibited, the continuous rearrangement of actin underlying microglial process movement should cease, and motility should decline.

Cytochalasin D, the most potent of the cytochalasins, was chosen over jasplakinolide, another inhibitor of actin treadmilling, because the latter can promote F-actin polymerization as well as stabilization,^37,38^ and apoptosis,^39^ and was preferred over latrunculin because the latter drug distorts cell shape far more than cytochalasin D.^40^ Nevertheless, we also applied jasplakinolide to some slices, as Bernstein and Bamburg^23^ stated that it reduced ATP use more effectively than cytochalasin D in neuronal cultures. In hippocampal slices, jasplakinolide has been used at concentrations from 0.1–10 µM^41,37^ without changing the electrophysiological properties of neurons.^41^ We used 1 µM.

Microtubule turnover was reversibly inhibited with 25 µM nocodazole (dissolved in DMSO), a non-cytotoxic microtubule-depolymerizing agent binding to beta-tubulin.^42^ This concentration was shown to be effective when bath-applied onto hippocampal slices without altering neuronal electrophysiological characteristics,^43^ and nocodazole depolymerises microtubules within minutes.^44^

To arrest fatty acid synthesis, we used 60 µM 5-(tetradecyloxy)-2 furoic acid (TOFA), which inhibits the acetyl-CoA carboxylase required to catalyse the carboxylation of acetyl-CoA.^45^ TOFA was dissolved in DMSO and 0.5% albumin to avoid precipitation. On adipocytes, 50 µM TOFA is efficacious within 15 min.^46^ Application of 60 µM TOFA for up to one hour does not alter neuronal viability.^45^

Anisomycin, an inhibitor of mRNA translation, was used at 20 µM (dissolved in DMSO) to inhibit protein synthesis. Anisomycin blocks protein synthesis within minutes at this concentration in hippocampal slices without affecting basal synaptic transmission.^47,48^

To inhibit the sodium–potassium ATPase, 1 mM ouabain was applied for 10 min in external solutions containing either 2 mM Ca^2+^ or 2 mM EGTA. To block all oxidative phosphorylation, 25 µM antimycin was applied for 20 min.

Equal percentages (see Supplementary Methods) of solvents or carrier proteins used were added to all the extracellular solutions in any particular experiment in order to rule out any confounding effects they may have. See Supplementary Methods for drug preparation and purchasing information.

### Oxygen recordings

**Figure 1.**
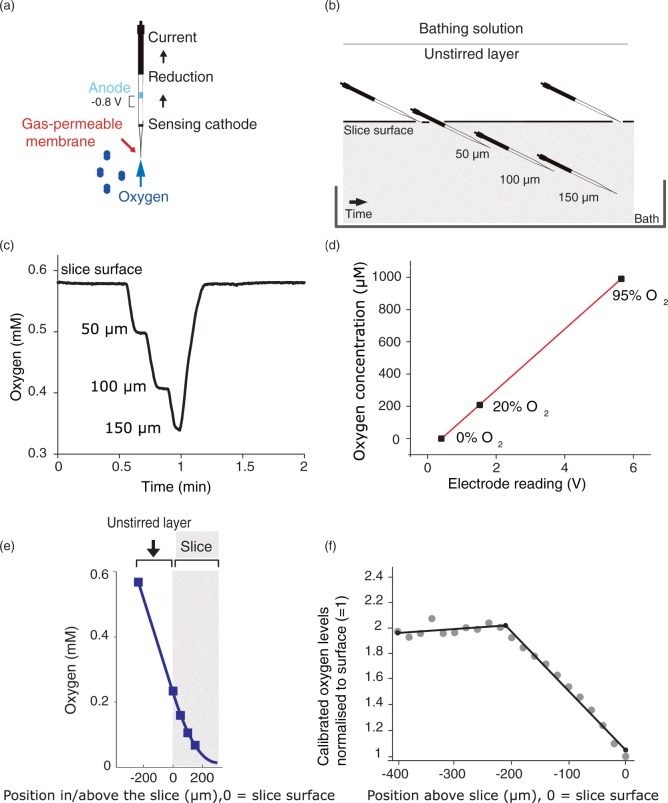
Measuring oxygen and calculating energy use in a brain slice. (a) A Clark-type oxygen sensor was used to measure oxygen concentration during the experiment (electrode schematic adapted from Unisense:^49^ for sensor information see Supplementary Methods). (b) Experimental outline. The oxygen sensor was placed at the surface (touching the tissue) of the hippocampal brain slice at the start of the experiment. After ∼10 min of baseline measurement, a depth profile of O_2_ concentration was obtained by moving the electrode along its own axis to generate a vertical depth of 50, 100, and 150 µm (the midpoint of the slice) into the slice (see Supplementary Methods). After obtaining the [O_2_] depth profile, the electrode was returned to the surface. Depth profiles measured with a greater spatial resolution, or with measurement points beyond the slice midpoint, did not affect the calculation of oxygen consumption through the slice (see Supplementary Figure 3). A specific blocker of an energy-consuming process was bath-perfused onto the slice for 7–20 min (see Materials and Methods and Supplementary Methods). A second [O_2_] depth profile was obtained at the end of the drug application, when the oxygen level had reached a plateau. After a 10–15 min recovery period and a third [O_2_] depth profile, 1 mM glutamate was applied to the slice and a final [O_2_] depth profile was obtained after oxygen levels stabilized after 3–5 min. Above the slice surface, oxygen diffuses through an unstirred layer but is not consumed. (c) Sample depth profile for [O_2_] through a hippocampal slice (CA1 region). The [O_2_] at the slice surface is lower than at the top of the static unstirred layer, where, in turn, the [O_2_] is lower than in the reservoir bubbled with 100% O_2_ (see (d)), as oxygen is lost to the air above the reservoir and through the perfusion tube walls (see Results). (d) Oxygen sensor calibration. Three bottles of distilled water were heated up to 37℃ and bubbled for at least 15 min with 0%, 20%, and 95% oxygen. The electrode was then inserted into the three solutions consecutively for a few seconds until a stable reading was obtained. Those readings were plotted against the dissolved oxygen concentrations corresponding to the different percentage saturation values for oxygen in water at 37℃, which were obtained from Henry's law as 0, 208, and 991 µM, respectively.^50^ Using the slope and intercept from the linear fit through these three points, a linear conversion to µM was then applied to the raw electrode output. (e) Oxygen measurements were taken at the end point of each depth step (blue dots). Measurements were fitted (see Materials and methods and Supplementary methods) with a modified diffusion equation (equation (1)) from the top of the unstirred layer to give *V*_max_, the maximum rate of oxidative phosphorylation at saturating [O_2_]. (f) The width of the unstirred layer was determined by moving the oxygen electrode upwards from the slice surface and calculating the break point of the [O_2_] profile between the unstirred layer and the bulk solution above it (see Supplementary Methods).

A Unisense Clark-type oxygen microsensor (OX-10), which generates a current proportional to the oxygen concentration,^49^ was used to measure the oxygen level at the slice surface (in the CA1 region of the hippocampus) and at three different depths in the slice before, during and after the application of a blocker of each non-signalling process (see Figure 1 and Supplementary Methods). Recordings were calibrated (Figure 1(d)) using solutions bubbled with known partial pressures of oxygen (converted to mM using Henry's law and the solubility^50^ of O_2_: see Supplementary Methods).

**Figure 2.**
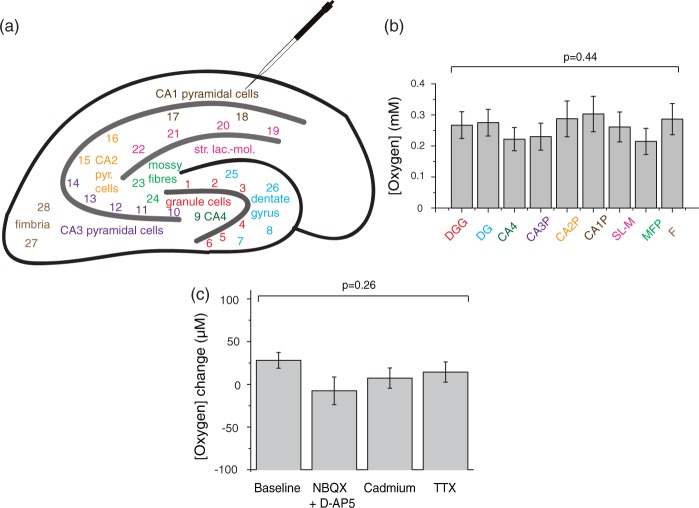
Baseline metabolic activity in a brain slice. (a) Constructing a hippocampal oxygen map of a P10 rat slice. The oxygen sensor was used to measure surface oxygen levels at each point on the schematic. The points were binned into nine distinct areas, colour-coded in (b). During the experiments reported subsequently in the paper, the electrode was placed in the CA1 region and advanced down through the slice into the stratum lacunosum-moleculare. (b) Mean ( ± s.e.m.) of oxygen concentration binned into the hippocampal regions shown in (a), n = nine slices, N = three animals. The following area measurements were binned: DGG (dentate gyrus granule cells) points 1–6, DG (dentate gyrus) 7–8 and 25–26, CA4 9, CA3P (CA3 pyramidal cells) 10–14, CA2P (CA2 pyramidal cells) 15–16, CA1P (CA1 pyramidal cells) 17–18, SL-M (stratum lacunosum-moleculare) 19–22, MFP (mossy fibre pathway) 23–24, and F (fimbria hippocampus) 27–28. No significant difference in oxygen level between hippocampal regions was found (*p* = 0.44). (c) Signalling related energy expenditure is negligible in a resting slice. Specific blockers of postsynaptic currents (and thus postsynaptic action potentials, 10 µM NBQX + 50 µM D-AP5, n = nine slices, N = four animals), presynaptic transmitter release and postsynaptic events (250 µM cadmium, n = 6, N = 2) or action potentials and synaptic events (1 µM TTX, n = 9, N = 9) were applied to different slices. Changes in oxygen level between the start of drug application and 15 min later were measured in those blockers and in a no-drug baseline condition (n = 9, N = 9). No blocker changed oxygen levels relative to control (p=0.26).

Since the electrode moved across the hippocampus when being lowered into the slice along its axis (starting from CA1 and usually not venturing beyond the stratum lacunosum-moleculare), we established that the baseline oxygen level was similar throughout the hippocampus by measuring the surface oxygen level at 28 points across 9 hippocampal regions (Figure 2(a) and (b)). We further determined the energy consumption on spontaneous electrical signalling in the hippocampal slice by blocking distinct signalling related processes (Figure 2(c)).

### Modelling oxygen consumption through the slice

The [O_2_] depth profiles obtained in each condition were used to model oxygen consumption throughout the slice, as in Hall et al.^51^ The depth profile data points, obtained during continuous flow of the solution superfusing the slice, were fitted with steady-state solutions of the following modified diffusion equation

(1) $$D\frac{\partial 2c}{\partial x2}=\frac{{\mathrm{cV}}_{\max}}{c+K_{m}}$$

where *D* = 1.54 × 10^−9^ m^2^/s is the diffusion coefficient of O_2_ in brain at 37℃,^52^ *c* is oxygen concentration, *x* is distance into the slice in µm, *V*_max_ denotes the maximum rate of oxidative phosphorylation at saturating oxygen concentration in mM/min, and *K_m_*=1 µM^53^ is the EC_50_ for oxygen activating oxidative phosphorylation. In equation (1), the left-hand side represents diffusion and the right-hand side represents the consumption of oxygen by mitochondria. This was solved using the *pdepe* function in MATLAB (the MathWorks; scripts available on request). At the bottom of the slice, we applied the boundary condition δ*c/*δ*x* = 0.

Above the surface of a slice, there is an unstirred layer of solution with no oxygen consumption, but only oxygen diffusion towards the slice from the bulk solution above^51,54^ with a diffusion coefficient for oxygen in water at 37℃ of 2.68 × 10^−9^ m^2^/s.^55^ Consequently, the oxygen concentration in the unstirred layer gradually approaches that of the bulk solution with greater distance from the membrane.^54^ In slices across all experimental conditions, the unstirred layer was measured after the experiment and incorporated into the fits of slice oxygen data in order to more accurately estimate changes in O_2_ consumption in the slice from changes in the O_2_ depth profiles (see Supplementary Methods).

Individual depth profiles, and depth profiles averaged over all slices, including the unstirred layer above the slice surface, were fitted with the non-linear least-square curve fitting function *lsqcurvefit* in MATLAB. All fits were evaluated by calculating the proportion of the sum of the squared residuals (difference between data and fit) explained by the fit (R^2^), and all fits in this paper had R^2^ > 0.95.

Most analyses involved measuring the [O_2_] profile through the depth of the slice; however, some experiments examined only the [O_2_] on the slice surface. Although the surface cell layer can be damaged by the slicing process^33,56^ up to a depth of ∼10 to 35 µm, this does not invalidate the use of surface [O_2_] measurements to assess the [O_2_] consumption of the slice, because the surface [O_2_] does not just reflect local metabolic activity, but reflects O_2_ use throughout the slice and thus changes when metabolic activity is altered (see plots in Figures 4(a) and (b), 5(a) and (b) and 6(a)).

**Figure 3.**
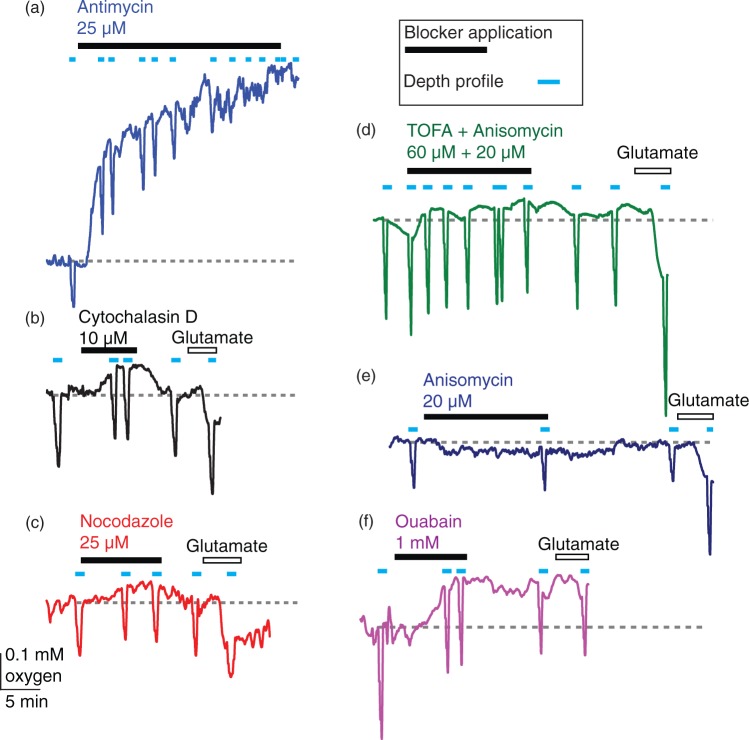
Sample traces for blockers of energy-consuming processes. Traces show oxygen levels on the slice surface interspersed with [O_2_] depth profiles (light blue bars). Oxygen levels rise when less oxygen is being consumed and fall when more oxygen is consumed (the grey dotted line is placed at the initial [O_2_] level at the slice surface for easier comparison). After ∼10 min of baseline (here only 5 min is shown before drug onset), the specific blocker of a non-signalling process was bath-perfused onto the slice (black bar), followed by 10–15 min of recovery and wash-in of 1 mM glutamate (open bar). Blocker application times are given in the Supplementary Methods. (a) antimycin stops all oxidative phosphorylation: near the end of the trace the variation of [O_2_] with depth is abolished, (b) cytochalasin D blocks actin treadmilling, (c) nocodazole inhibits microtubule turnover, (d), TOFA arrests lipid synthesis, (e) anisomycin blocks protein synthesis and (f) ouabain inhibits the sodium–potassium ATPase (no calcium in the external solution).

**Figure 4.**
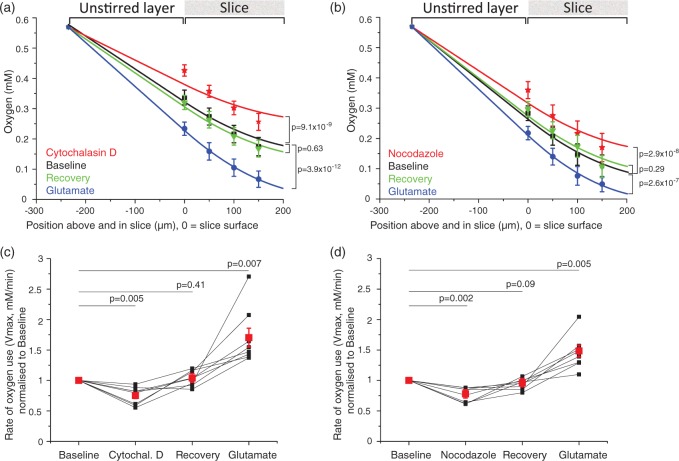
Actin cytoskeleton treadmilling accounts for about a quarter of resting energy use, and microtubule turnover uses a similar fraction of the brain's energy. (a), (b) Average oxygen concentration (mM ± s.e.m.) depth profiles for each condition (black = baseline, red = 10 µM cytochalasin D in (a) and 25 µM nocodazole in (b), green = recovery, blue = 1 mM glutamate), for block of actin ((a), n = eight slices, N = six animals) and microtubule ((b), n = 8, N = 4) turnover. Data were fitted with equation (1) from the surface to the bottom of the slice, and with equation (1) but without the oxygen consumption term across the unstirred layer to the slice surface. The fit gives V_max_, the maximum rate of oxygen use. (c), (d) Averaged V_max_ ± s.e.m. (red dots) and individual V_max_ values (black dots, normalised to baseline V_max_ (=1)) for block of actin ((c), n = 8) and microtubule ((d), n = 8) turnover. The average fractional rate of energy consumption relative to baseline was calculated from the average of individual fits as being 75% during actin treadmilling inhibition and 78% during microtubule turnover block.

**Figure 5.**
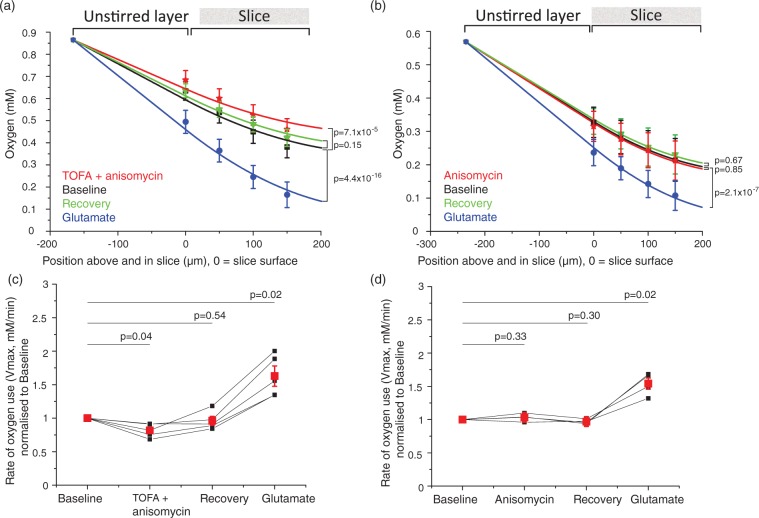
Lipid and protein synthesis together account for about 18% of O_2_ use, but O_2_ use on protein synthesis alone is too small to be measured. (a), (b) Average oxygen concentration ± s.e.m. across depth profiles per condition (black = baseline, red = 60 µM TOFA + 20 µM anisomycin in (a) or 20 µM anisomycin in (b), green = recovery, blue = 1 mM glutamate) for block of lipid and protein synthesis ((a), n = five slices, N = two animals) and protein synthesis alone ((b), n = 4, N = 4). (c), (d) Average V_max_ ± s.e.m. (red dots) and individual V_max_ (black dots, normalised to baseline V_max_ (=1)) for block of lipid and protein synthesis ((a), n = 5) and protein synthesis alone ((b), n = 4). The average energy consumption was 82% of the control value when lipid and protein synthesis were blocked. No change could be detected when protein synthesis alone was inhibited.

**Figure 6.**
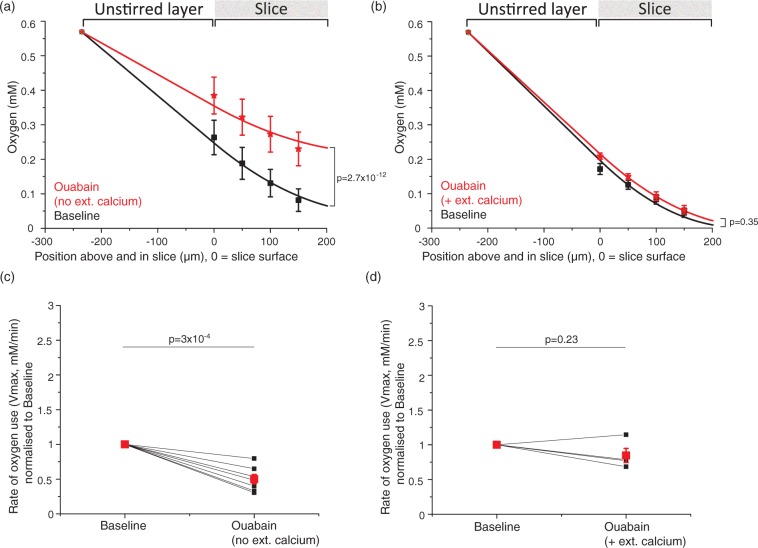
Block of the sodium–potassium ATPase nearly halves oxygen use in the absence of external calcium. (a), (b) Averaged oxygen concentration ± s.e.m. across depth profiles per condition (black = baseline, red = 1 mM ouabain) for inhibition of the sodium–potassium pump in the absence ((a), n = seven slices, N = two animals) and presence ((b), n = 4, N = 4) of external calcium. Slices did not recover after ouabain application and did not react to glutamate (data not shown). (c), (d) Averaged V_max_ ± s.e.m. (red dots) and individual V_max_ values (black dots, normalised to baseline V_max_ (=1)) for inhibition of the sodium–potassium pump in the absence ((c), n = 7) and presence ((d), n = 4) of external calcium. In ouabain, the average energy consumption was 50% of the control value in the absence of external calcium, an effect masked by the presence of calcium (see Results and Discussion).

### Statistics

Data are shown as mean ± standard error of the mean (s.e.m.). After confirming that the data were normally distributed using the Kolmogorov–Smirnov test, one-way or two-way ANOVAs (repeated measures where appropriate) or paired or one-sample t-tests were used to compare means, and data were corrected for multiple comparisons with Dunnett's post hoc test or a modified Holm–Bonferroni correction (see Supplementary Methods). Degrees of freedom are reported in brackets after the t or F statistic, respectively.

## Results

### Baseline hippocampal oxygen levels are uniform in hippocampal slices

The hippocampus comprises different functional areas which might have different baseline energy uses. The oxygen electrode moved laterally through the slice when lowered along its axis so, although it mostly remained in the CA1 region, we investigated whether regional differences in baseline [O_2_] might affect our results. We tested this by measuring the oxygen level at the slice surface at 28 points across 9 hippocampal regions (Figure 2(a) and (b)). No significant difference in [O_2_] across regions was detected (F(8,72)=0.44, *p* = 0.89, n = nine slices), suggesting no significant difference in O_2_ use between hippocampal areas. This implies that moving the oxygen electrode across the slice during the process of lowering it into the slice would not have an effect on the measured [O_2_].

### No O_2_ use associated with spontaneous activity can be detected in brain slices

Most brain energy is used on synaptic and action potentials,^1^ and this can be detected as changes of [O_2_] level in response to electrical stimulation in hippocampal slices.^51^ However, endogenous synaptic activity is less in brain slices than in the brain, because long-range connections are disrupted by the slicing. We investigated oxygen use evoked by spontaneous electrical activity by blocking either postsynaptic currents (using 10 µM NBQX + 50 µM D-AP5, n = nine slices), presynaptic transmitter release and postsynaptic events (using 250 µM cadmium, n = six slices), or action potentials and synaptic events (using 1 µM TTX, n = nine slices). None of these manipulations significantly changed the oxygen level at the slice surface (F(3,29)=1.4, *p* = 0.26, Figure 2(c)), suggesting that ongoing electrical activity in the slice is too weak to be detected from its O_2_ consumption. The absence of spontaneous signalling activity facilitated selective measurement of the O_2_ use of non-signalling processes, but also implies that the percentage of O_2_ use that we measure for non-signalling tasks (below) would be a smaller percentage of total brain O_2_ consumption in vivo, where synaptic and action potentials consume more energy (see Discussion).

### Unstirred layer parameters

In the following experiments, we fit measurements of O_2_ concentration throughout the slice and the unstirred layer above the slice to obtain a value for the rate of oxygen consumption in the slice. We incorporated the value of [O_2_] at the top of the unstirred layer (which corresponds to the oxygen level in the bulk solution) in order to more accurately estimate changes in O_2_ consumption in the slice from changes in the O_2_ depth profiles (see Materials and Methods and Supplementary Methods). The unstirred layer width averaged across the cytochalasin D, nocodazole, ouabain, and anisomycin conditions in 17 slices was 235 ± 10 µm, and the [O_2_] in the bulk solution at the top of the unstirred layer was 0.57 ± 0.05 mM (less than the 1.04 mM in the solution reservoir bubbled with 100% O_2_ due to O_2_ loss to the air above the reservoir and through the perfusion tube walls). Neither the width of the unstirred layer nor the [O_2_] at the top of the unstirred layer differed significantly between these conditions (F(3,13)=2.8, *p* = 0.08 and F(3,13)=2.2, *p* = 0.13, respectively, n = 17 slices).

For experiments containing albumin as a carrier protein for the lipid synthesis blocker TOFA, the unstirred layer parameters were significantly different. The unstirred layer width in four slices was 165 ± 21 µm, and the [O_2_] at the top of the unstirred layer was 0.86 ± 0.45 mM. In the bulk solution outside the unstirred layer (unaffected by the slice's oxygen consumption), the [O_2_] above 17 slices superfused with solution not containing albumin was 0.59 ± 0.04 mM, but was 0.92 ± 0.02 mM when external solutions containing albumin were used. Therefore, the raised baseline [O_2_] in experiments using solutions containing albumin (described below) is an effect of a greater saturation of the bulk solution with oxygen, which may come about because the albumin foam formed on the surface of solution bubbled with gas impedes the loss of the bubbled O_2_ to the air above.

### Blocking all oxidative phosphorylation raises the [O_2_] to the bulk solution level

In order to confirm that our method of measuring [O_2_] in a brain slice with an oxygen electrode does indeed reflect changes in cellular respiration, we verified that blocking all oxidative phosphorylation in a brain slice elevated [O_2_] at the slice surface to bulk solution levels. Application of 25 µM antimycin, a respiratory chain inhibitor, increased the oxygen concentration at the slice surface within one to two minutes of application. After 15 min, the oxygen concentration at the surface of the slice (0.60 ± 0.02 mM) was not significantly different from that in the bulk solution of the bath (which in these experiments was 0.66 ± 0.01 mM, t(3)=1.88, *p* = 0.16, n = slices slices) and was much larger than the surface oxygen level before drug application (0.24 ±0.06 mM). Consistent with this, the variation of [O_2_] with depth was abolished within minutes when oxygen consumption throughout the whole slice ceased (Figure 3(a)).

### Oxygen level changes after block of non-signalling processes

Figure 3(b) to (e) shows sample traces for oxygen level changes at the slice surface for each non-signalling process that was blocked and the recovery of the oxygen level after the blocker was removed, while Figure 3(e) shows oxygen level changes after block of the sodium pump. At the end of each experiment, to check the health of the slice, 1 mM glutamate was applied to activate a cation influx through ionotropic receptors and thus increase oxygen consumption. For all drugs, oxygen level was measured as a function of depth in the slice, and these depth profiles were used to calculate the rate of oxygen consumption through the slice, by solving a modified diffusion equation (see Materials and methods and Supplementary methods). These rates of oxygen consumption are quantified in Figures 4 to 6.

### Blocking actin or microtubule cycling reduces oxygen consumption

To investigate the effect on energy consumption of blocking actin treadmilling, 10 µM cytochalasin D was applied to the slice to arrest actin polymerisation. At the end of the cytochalasin D application (Figure 3(b)), the oxygen level at the slice surface was elevated from 0.33 ± 0.02 mM to 0.43 ± 0.02 mM (n = 8), suggesting that some slice oxygen consumption is due to actin treadmilling. On removing cytochalasin D, the oxygen level recovered to 0.32 ± 0.02 mM (Figure 4(a)). Subsequently, applying glutamate lowered the surface oxygen level to 0.23 ± 0.02 mM, reflecting oxygen consumption to fuel synaptic depolarisation. A two-way repeated measures ANOVA for treatment (baseline, cytochalasin D, recovery, glutamate) and position in the slice (surface, −50 µm, −100 µm, −150 µm) showed a main effect of condition on oxygen level across all depths used in the depth profile (F(3,21)=33.9, *p* =2.1 × 10^−8^, n = eight slices). With Dunnett's post-hoc test, the oxygen levels in cytochalasin differed significantly from control (t(21)=8.42, *p* = 9.1 × 10^−9^), and there was no difference in the oxygen levels between the baseline and recovery conditions (t(21)=1.01, *p* = 0.63). Glutamate application also significantly changed the oxygen levels relative to baseline (t(21)=10.81, *p* = 3.9 × 10^−12^, n = eight slices). Similarly, when 1 µM jasplakinolide, another actin cycling blocker that can, however, also promote polymerization,^38^ was applied, the surface oxygen level rose from 0.23 ± 0.03 mM to 0.28 ± 0.02 mM (t(6)=5.56, *p* = 0.004) in three slices.

We then modelled the rate of oxygen consumption through the slice. After the [O_2_] depth profiles were fit (Figure 4(a)) with the modified diffusion equation (equation (1)) incorporating the unstirred layer, the resulting values of V_max_, the maximum rate of oxidative phosphorylation at saturating [O_2_], were normalised to the initial control value for each slice. On average, the *V*_max_ in cytochalasin D was 0.75 ± 0.05 of that in the initial control condition (Figure 4(c), t(7)=−4.8, *p* =0.005, n = eight slices). On removing cytochalasin D, *V*_max_ recovered to 1.03 ± 0.04 of the initial control value (t(7)=0.88, *p* = 0.41). In glutamate, the average *V*_max_ rose to 1.70 ± 0.1 of the initial control value (Figure 4(c), t(7)=4.29, *p* = 0.007, n = eight slices). These results suggest that a significant fraction of the slice's resting energy budget, 25%, is spent on actin cycling. Two-photon imaging of ongoing microglial motility before and after the application of cytochalasin D confirmed that the drug rapidly and effectively inhibited microglial movement and therefore actin cycling (Supplementary Figure 1(c), n = 15 cells from five slices).

In order to inhibit microtubule turnover, 25 µM nocodazole, a microtubule-depolymerising agent was perfused onto the slice. Microtubule turnover also contributes to resting brain slice oxygen use, as applying nocodazole (Figure 3(c)) raised the oxygen level at the slice surface from 0.28 ± 0.02 mM to 0.36 ± 0.03 mM. This recovered to 0.30 ± 0.03 mM on removing the nocodazole (Figure 4(b)), while glutamate lowered the surface oxygen level to 0.22 ± 0.02 mM (n = 8). A two-way repeated measures ANOVA again showed a main effect of condition on oxygen level across all depths in the slice (F(3,21)=37.8, *p* = 1.2 × 10^−8^, n = eight slices). Oxygen levels across depth in nocodazole differed significantly from baseline (Figure 4(b), t(21)=8.03, *p* = 2.9 × 10^−8^) and the surface oxygen level after recovery from nocodazole application was indistinguishable from baseline (t(21)=1.59, *p* = 0.29). Similarly, glutamate application significantly lowered [O_2_] relative to the control condition (t(21) = 7.28, *p* = 2.6 × 10^−7^, n = eight slices).

As in the previous experiment, these data were used to model oxygen consumption through the slice. When microtubule turnover was blocked with nocodazole (Figure 4(d)), the *V*_max_ derived from the [O_2_] depth profiles was reduced to 0.78 ± 0.04 (t(7) = −5.6, *p* =0.002, n = eight slices) of the control value, and recovered to 0.96 ± 0.03 of the control value on removing nocodazole (t(7) = −1.9, *p* = 0.09). In glutamate, *V*_max_ increased to 1.48 ± 0.10 of the baseline value (t(7)=4.6, *p* = 0.005, n = eight slices). Surprisingly, therefore, microtubule turnover also accounts for a substantial 22% of the slice's baseline oxygen use. After the slices recovered from either actin or microtubule turnover inhibition, activating glutamate receptors throughout the slice by superfusing glutamate increased the energy use by 50–70%. The magnitude of the V_max_ change after glutamate application was not significantly different in the cytochalasin D versus the nocodazole condition (t(14) = −0.4, *p* = 0.67).

Since excitatory synaptic currents are thought to consume most ATP in the brain,^9^ we were concerned that our estimates of the energy use on cytoskeletal recycling could be confounded if the drugs used affected the frequency of excitatory synaptic currents (see Supplementary Figure 1(a) and (b)). We therefore whole-cell patch-clamped area CA1 pyramidal cells and monitored spontaneous EPSCs while applying either cytochalasin D or nocodazole (see Supplementary Methods). Neither drug significantly altered the number of EPSCs occurring during the last 6 min in each condition (F(2,14)=1.69, *p* = 0.23 for cytochalasin D and F(2,8)=0.56, *p* = 0.59 for nocodazole, both n = eight slices).

### The energy used on lipid and protein synthesis

We next investigated the contributions of phospholipid and protein synthesis to the slice's energy expenditure. Previous estimates of the energy consumption of protein synthesis are unanimously very low, at ∼2% of the total energy budget,^1,14,29^ but vary between 2% and 25% for lipid metabolism.^14,30,31,57^ We first applied blockers of both phospholipid synthesis (60 µm TOFA) and protein synthesis (20 µM anisomycin) together. In the experiments containing TOFA, serum albumin had to be present in the external solutions to prevent precipitation of the drug. Albumin increased the concentration of oxygen in the solutions (see above), which altered oxygen levels at the slice surface (F(2,16)=12.74, *p* =0.006, n = four slices). Application of 0.5% albumin (together with 0.55% DMSO) reversibly raised the oxygen level at the surface of the resting slice from 0.34 ± 0.03 mM to 0.56 ± 0.06 mM (t(6)=10.1, *p* =0.8 × 10^−5^). The surface oxygen level recovered to 0.34 ± 0.05 mM after albumin was washed off (t(6)=41, *p* = 0.88, n = four slices). As albumin was added to all solutions in the lipid synthesis block experiments, the baseline oxygen level was uniformly raised throughout these experiments. To test whether, by altering the oxygen level in the superperfused solution, albumin changed the oxygen consumption of the slice in control conditions, we assessed the total oxygen consumption as being proportional to the amount of O_2_ diffusing through the unstirred layer towards the slice. This flux is proportional to the difference between the oxygen level at the top of the unstirred layer (a mean value for which was averaged over all slices) and at the slice surface (measured for each individual slice), divided by the width of the unstirred layer (averaged over all slices). This parameter did not differ between experiments without albumin in the external solution (1.22 ± 0.07, n = 31 slices) and those using external albumin (1.36 ± 0.26, n = five slices, t(34) = −0.7, *p* = 0.49).

After lipid and protein synthesis were blocked using 60 µM TOFA and 20 µM anisomycin (Figure 5(a)), the oxygen level at the slice surface rose from 0.63 ±0.04 mM to 0.68 ± 0.04 mM (t(12)=6.2, *p* = 7.0 × 10^−5^). On removal of the drugs, the [O_2_] recovered to 0.64 ± 0.03 mM (t(12)=2.06, *p* = 0.14), and on applying glutamate it fell to 0.49 ± 0.05 mM (t(12)=17.01, *p* = 4.4 × 10^−16^). An overall two-way repeated measures ANOVA showed a main effect of condition on oxygen levels across depths (F(3,12)=79.1, *p* = 3.75 × 10^−8^, n = five slices).

We then blocked protein synthesis alone with 20 µM anisomycin (Figure 5(b)) to isolate its contribution to the slice's oxygen consumption. There was no significant change in oxygen level at the slice surface between baseline (0.32 ± 0.05 mM), anisomycin (0.32 ± 0.05 mM, t(9)=0.66, *p* = 0.85) and recovery (0.33 ± 0.04 mM, t(9)=0.96, *p* = 0.67). However, [O_2_] again fell significantly in glutamate to 0.23 ± 0.04 mM (t(9)=10.98, *p* = 2.14 × 10^−7^).

When modelling the rate of oxygen consumption through the slice, the *V*_max_ for O_2_ usage after inhibition of both lipid and protein synthesis (Figure 5(c)) fell to 0.82 ± 0.05 of the control value (t(4)=−4.1, *p* = 0.04), recovered to 0.96 ± 0.07 of the control value on removing the blockers (t(4) = −0.66, *p* = 0.54), and increased to 1.63 ± 0.15 of the control value (t(4)=4.61, *p* = 0.02) in glutamate. For protein synthesis block alone (Figure 5(d)), the *V*_max_ for O_2_ usage in anisomycin relative to baseline was 1.03 ± 0.03, not significantly different from the control value (t(3)=1.15, *p* = 0.33). After removing the drug, the recovery value was 0.97 ± 0.02 of the control value (t(3) = −1.9, *p* = 0.30), while *V*_max_ in glutamate rose to 1.54 ± 0.08 of the control value (t(3)=6.39, *p* = 0.02). From the data above, it can therefore be estimated that lipid and protein synthesis together require 18% of the brain's resting O_2_ use. Lipid synthesis likely account for most of this figure, as protein synthesis uses too little of the slice's resting energy to be detected with the oxygen electrode.

### Without external calcium, the Na^+^/K^+^ pump accounts for 50% of energy use

Most brain ATP use is thought to be on the pumping out of ions that enter neurons to generate synaptic or action potentials, or that enter at the resting potential.^9^ This pumping is mainly carried out by the sodium pump. To examine the fraction of energy expended on sodium pumping in brain slices, we applied the pump blocker ouabain (1 mM). These experiments were performed both in the presence and absence of external calcium, because blocking the pump is expected to lead indirectly to a rise of [Ca^2+^]_i_ which can increase energy consumption (see Discussion). For example, while Shibuki^58^ found that ouabain decreased oxygen consumption in unstimulated neurohypophysis slices whether or not calcium was present, Ruščák and Whittam^59^ found that blocking the Na^**+**^/K^+^ pump only decreased O_2_ consumption in cortical slices when using Ca^2+^-free external solutions, and instead found an increase in O_2_ consumption when the external solution contained Ca^2+^. Because of these varying results, we measured the effect of blocking the sodium pump on the [O_2_] profile both in the presence and absence of external calcium.

After ouabain application in the absence of external calcium, the oxygen level at the slice surface rose from 0.26 ± 0.05 mM to 0.38 ± 0.05 mM (Figure 6(a); recovery and effect of glutamate are not shown as the effect of ouabain was irreversible). The main effect of ouabain on oxygen levels was significant at all depths in the slice (F(3,18)=17.86, *p* = 1.2 × 10^−5^, n = seven slices). Oxygen levels in the presence of ouabain differed significantly from those in control conditions (t(18)=11.55, *p* = 2.7 × 10^−12^). In contrast, with external calcium present, the oxygen level at the slice surface rose from 0.17 ± 0.02 mM to 0.20 ± 0.01 mM (Figure 6(b)) when ouabain was applied, which was not significant (F(3,6)=1.32, *p* = 0.35, n = four slices). This is presumably because the pump block raises [Ca^2+^]_i_ which increases energy consumption (see Discussion).

When modelling the rate of oxygen consumption through the slice, the average *V*_max_ for oxygen usage in ouabain without external calcium (Figure 6(c)) was 0.50 ± 0.07 of the control value without calcium (t(6) = −7.4, *p* = 3×10^−4^, n = seven slices). With external calcium present (Figure 6(d)), the *V*_max_ after ouabain application was not significantly different from baseline (0.85 ± 0.10 of the control value, t(3) = −1.5, *p* = 0.23, n = four slices). Without external calcium, blocking the sodium pump therefore approximately halved the resting slice's energy use, whereas no significant change in oxygen consumption could be detected when ouabain was applied in the presence of external calcium.

## Discussion

Here, we have presented the first experimental data obtained with a single method investigating the relative contributions of the main non-signalling processes to the brain's energy budget. This was done by inhibiting these processes in young rat hippocampal slices and measuring changes in oxygen consumption with a Clark-type oxygen sensor. Surprisingly, the actin and microtubule cytoskeletons contribute almost equally to a large (∼47%) fraction of the brain slice's resting energy budget (Figure 4). The O_2_ use on actin turnover (25%) is considerably lower than previous suggestions^23^ that half of the energy use was spent on actin turnover alone, but is much higher than our previous modelling suggested.^24^

Lipid synthesis accounts for most of the remaining non-signalling energy use (∼18% when blocked together with protein synthesis), but blocking protein synthesis did not detectably change energy use (Figure 5). These results are consistent with previous estimates that protein synthesis accounts for only ∼1.3% of the brain's total energy use.^1,29^ Purdon and Rapoport^31,57^ calculated that up to 25% of the brain's energy use might be spent on phospholipid metabolism, although only 5% of the total energy budget was estimated to be spent on the turnover of fatty acids within phospholipids, with the rest allocated to maintaining the phosphorylation state of phospholipids (12%) and maintaining asymmetries in the phospholipid bilayer (7.7%).

As in previous experiments in vivo^12^, inhibiting the sodium–potassium pump approximately halved the total energy expenditure in our brain slices. However, as in cortical slices,^58,59^ this pronounced reduction in oxygen consumption was seen only when external calcium was absent, and the decrease in oxygen consumption was inhibited when calcium was present in the external solution (Figure 6). It is known that, with extracellular calcium present, blocking the sodium pump with ouabain induces a rise of intracellular [Ca^2+^]_i_, due to Na^+^ gradient rundown and subsequent reversal of the Na^+^/Ca^2+^ exchanger^60^. This may, in turn, increase the activity of oxygen-consuming processes (such as Ca^2+^-ATPase activity to remove the extra Ca^2+^) as well as increasing oxidative phosphorylation,^61^ which could occlude the decrease in oxygen consumption produced by sodium–potassium pump inhibition.

The contributions to O_2_ consumption of the sodium pump (50%), cytoskeletal turnover (47%), and protein and lipid synthesis (18%) that we have estimated sum to slightly more than 100%. However, the sodium pump contribution was calculated in the absence of calcium, and furthermore the experimental results presented here need to be considered with several caveats.

First, we are assuming that oxygen consumption is proportional to energy use, because under normal conditions glucose is essentially completely oxidised in the brain by the sequential operation of glycolysis and oxidative phosphorylation.^4,5^ However, we cannot exclude the possibility that, in our brain slices, glycolysis accounts for a larger proportion of ATP generation than in the normal brain. For example, microglia, the brain's immune cells, switch their energy production mechanism from oxidative phosphorylation to glycolysis in hypoxia^62^, and the relative rates of these processes in astrocytes and neurons^63^ could differ in slices and in vivo. However, in these experiments, the slices were superfused with 100% oxygen and thus were not limited by oxygen availability.

Second, there is a limit to the resolution of oxygen measurements using Clark-type oxygen sensors. Baseline recordings of brain oxygen levels show fluctuations of ∼25 µM oxygen over 15 min (Figure 2(c), baseline). The noise that this introduces, together with block of the sodium–potassium pump being performed in the absence of external calcium, could account for the sum of the contributions of all processes exceeding 100%.

How can our estimates of non-signalling energy use in brain slices be extrapolated to the awake brain? Oxidative phosphorylation is lower in unstimulated brain slices than in vivo, since there is little spontaneous neuronal activity in slices (Figure 2(c)). The resting oxygen consumption in our experiments (in P10 rats) was 0.47 ± 0.03 mM/min at 37℃, while 0.7 mM/min was found previously in coronal brain slices of P21 rats at 35℃^51^, 0.8 mM/min in cultured cerebellar slices of P8 rats, and 1.3 mM/min in acute cerebellar slices of P10 rats, both at 37℃.^64^ However, oxygen consumption is higher in vivo: in anaesthetized rats, the CMRO_2_ at 37℃ is approximately 1.8 mM/min,^65^ while Sokoloff et al.^66^ estimated glucose consumption in the grey matter of conscious adult rats to be ∼1 mM/min, implying an oxygen consumption of 6 mM/min. In awake humans, the whole-brain averaged CMRO_2_ is around 1.3 mM/min.^67^ The much lower oxygen consumption in our data (0.47 ± 0.03 mM/min under baseline conditions) presumably reflects the young age of the animal and the presence of little neuronal activity in the slice (Figure 2(c)) which is a major contributor to oxygen use.^51,63,68–71^ Interestingly, unlike our data in Figure 2(c), Huchzermeyer et al.^68^ found a rise in oxygen level in organotypic slices following the application of TTX, implying that sufficient spontaneous activity was occurring to generate detectable O_2_ use. Whether this reflects the use of organotypic slices or of an interface chamber^69^ by Huchzermeyer et al.,^68^ as opposed to the submerged acute slices used in this study, is unclear.

If the non-signalling energy use were the same independent of age, brain location or level of neuronal activity, then the percentage contributions of non-signalling energy processes to the total energy budget would be significantly smaller in vivo than we calculate above. However, non-signalling processes are not completely uncoupled from signalling activity: for instance, actin turnover is regulated by neuronal activity, with faster treadmilling after block of neuronal activity and reduced motility due to actin stabilisation after NMDA application.^19,72^ This would imply that the energetic cost of actin cycling in brain slices, with little neuronal activity, is higher than in the intact brain.

It is also likely that non-signalling processes in more mature animals have a different energy demand from those at the early developmental stage used here (P10) when cell processes are still being extended. Energy metabolism as a whole changes: at P10, rodents may lack full capabilities for oxidative phosphorylation, which might explain the lower baseline *V*_max_ in our slices compared to those from older animals,^50^ and neuronal function is more resistant to oxygen deprivation than in mature rats.^73^ In the case of the actin cytoskeleton, developmental changes occur: motility in highly dynamic dendritic spines decreases significantly between P10 (a peak time for synaptogenesis) and P20 in cortical mouse slices.^74^ Total actin levels in the brain also peak at an early stage of development and later decline.^75^ This would again imply that actin recycling uses less energy in vivo than in our brain slices. The pattern of microtubule spatial organisation also shifts during early development: uniformly high-polymerisation rates throughout neuronal axons and dendrites in the first days of development give way to more stable proximal regions in older cultured neurons, while more dynamic distal regions maintain high polymerisation rates.^76^ Furthermore, de novo phospholipid and protein synthesis both decline with age.^77^ Non-signalling processes are thus developmentally regulated, and it would be valuable to repeat the experiments presented here at different stages of development. Here, we have presented data at P10, an age of rapid synapse restructuring and synaptogenesis.^78^ It would be interesting to investigate whether the energetic cost of actin cycling decreases after ∼P20, when synapses have stabilised and less cytoskeletal rearrangement is needed. In addition, it would be interesting to test for regional differences in the non-signalling energy budget.

In summary, contributions of non-signalling processes to the brain's energy budget can be significant, but are probably dynamic and likely to change during development or with changes in the brain's functional state. Pathology will also alter non-signalling processes and their energetic consumption. Alzheimer's disease (AD), in which energy supply is impaired, affects actin and microtubule turnover.^79,80^ Cofilin, an actin-binding protein, may link cytoskeletal aberrations to mitochondrial impairments characteristic not only of AD, but also of related pathologies such as Parkinson's and Huntington's disease.^81^ Understanding how non-signalling processes contribute to the brain's energy budget is therefore important. As a first step, we have found that both actin and microtubule cytoskeleton turnover are surprisingly significant energy drains in the healthy developing brain, with lipid synthesis close behind. In contrast, protein synthesis is energetically inexpensive.

## Supplementary Material

Supplementary material

## Supplementary Material

Supplementary material
