# Supplementary material for: Non-signalling energy use in the developing rat brain
Source: J Cereb Blood Flow Metab. 2016 Jul 20;37(3):951–66. doi: 10.1177/0271678X16648710 (PMC5322833; doi:10.1177/0271678X16648710)
Supplement: Supplementary material [file JCBFM-0026-16-ORIG.R1_710.docx]

**Supplementary Figures**


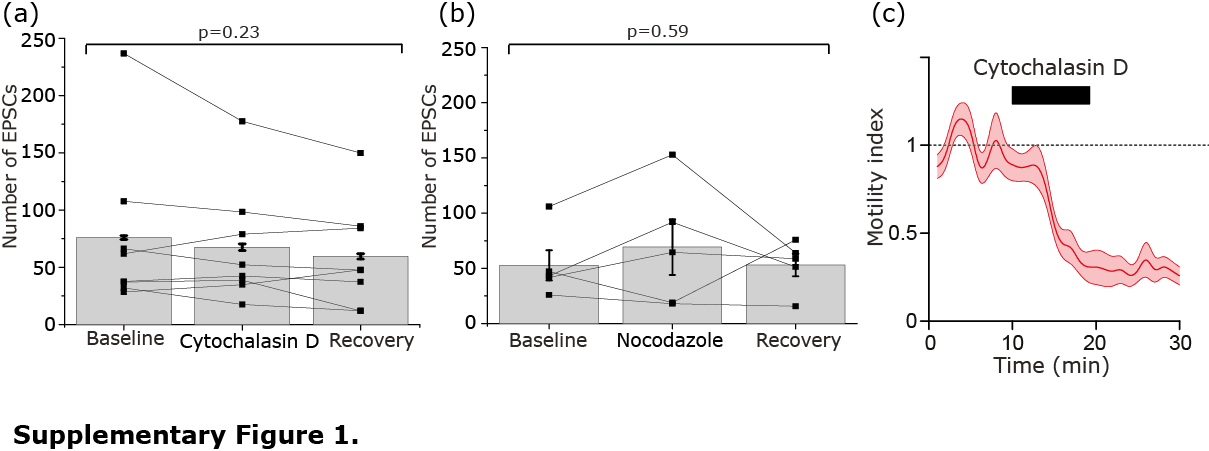


**Supplementary Figure 1.**

The effect of inhibiting the actin or microtubule cytoskeleton on neuronal properties and microglial motility. (a) Patch-clamping pyramidal neurons (at a depth of ~10-30 µm below the slice surface) and applying 10 µM cytochalasin D for 10 mins showed that spontaneous electrical activity of the cell (spontaneous EPSCs) did not change during drug application (see Results). The number of spontaneous EPSCs was counted for the last 6 mins of the baseline period, of the 10 min drug application period (allowing 4 mins for the drug to wash in and become effective), and of the 10 min recovery period, respectively (n=8). There was no effect of condition on the number of spontaneous EPSCs (p=0.23). (b) Similarly, applying 25 µM nocodazole for 10 mins onto pyramidal neurons had no effect on the number of spontaneous EPSCs (p=0.59, n=8). Consistent with previously published values, the mean input resistance of the patch-clamped pyramidal neurons was 260±34 MΩ (n=16 cells),^1^ and the mean resting membrane potential was -73.0±1.4 mV including the junction potential of -14 mV, in the range typical for these neurons.^2^ Patch-clamped cells also showed normal voltage-dependent sodium currents in response to voltage steps to potentials between -20 and +40 mV. From the baseline conditions in (a) and (b), the mean frequency of the spontaneous EPSCs can be calculated as 0.18±0.03 Hz, which is at the lower end of the range found previously in CA1 neurons in rats of a similar age^3^, and significantly less than the higher spontaneous EPSC frequency seen in CA1 pyramidal neurons in kainate-induced epilepsy^1^ (increased from ~0.45 Hz in control conditions to ~1.2 Hz). These findings suggest that the neurons in our slice preparation were healthy and their spontaneous EPSCs were not generated by any damage or epileptic activity caused by the slicing procedure. (c) Microglial motility, defined as the sum of pixel-wise microglial process extensions and retractions (normalised by extensions and retractions over the baseline condition: see Supplementary Methods), declines rapidly after the application of 10 µM cytochalasin D (black bar; n=15 cells from 5 slices, drug application time 9 mins).


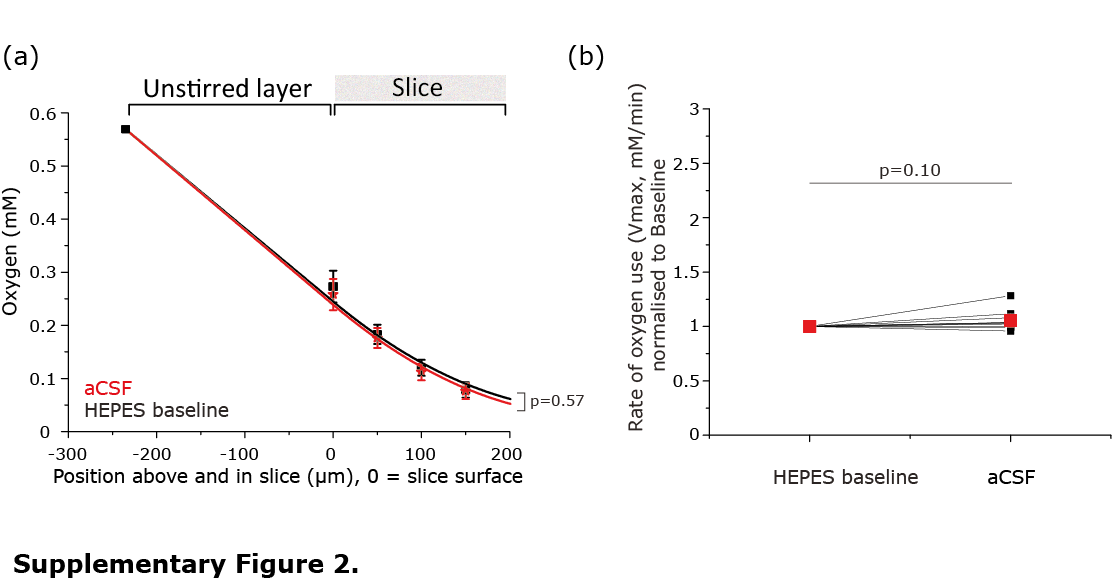


**Supplementary Figure 2.**

Resting oxygen levels and oxygen consumption across the brain slice did not differ whether the slice was perfused with a HEPES-buffered or a sodium bicarbonate-buffered (aCSF) external solution. (a) Average oxygen concentration + s.e.m. across depth profiles for different external solutions (black = HEPES baseline, red = aCSF) in 5 slices. No difference between the oxygen levels in the aCSF and the HEPES conditions across the different depths in the slice could be detected (F(1,9)=0.33, p=0.57, n=10 depth profiles per condition). The mean oxygen level at the slice surface was 0.27±0.03 mM in the HEPES condition and 0.26±0.03 mM in the aCSF condition. Each external solution was applied for 5 minutes before the depth profile was obtained. (b) Averaged V_max_ + s.e.m. (red dots) and individual V_max_ values (black dots, normalised to the V_max_ of the HEPES baseline) for the aCSF external solution condition. Oxygen consumption through the slice did not differ between the aCSF and HEPES conditions: on average, the V_max_ in the aCSF condition was 1.06±0.09 of that in the initial HEPES baseline condition (n as in (a), t(9)=1.9, p=0.10). The mean V_max_ was 0.48±0.04 mM/min when the external solution was HEPES-based, and 0.51±0.06 mM/min when it was bicarbonate-based (not significantly different, p=0.19; n as in (a)), which is similar to the baseline V_max_ of 0.47±0.03 mM/min found previously across all experiments using HEPES-based external solutions (n=39).


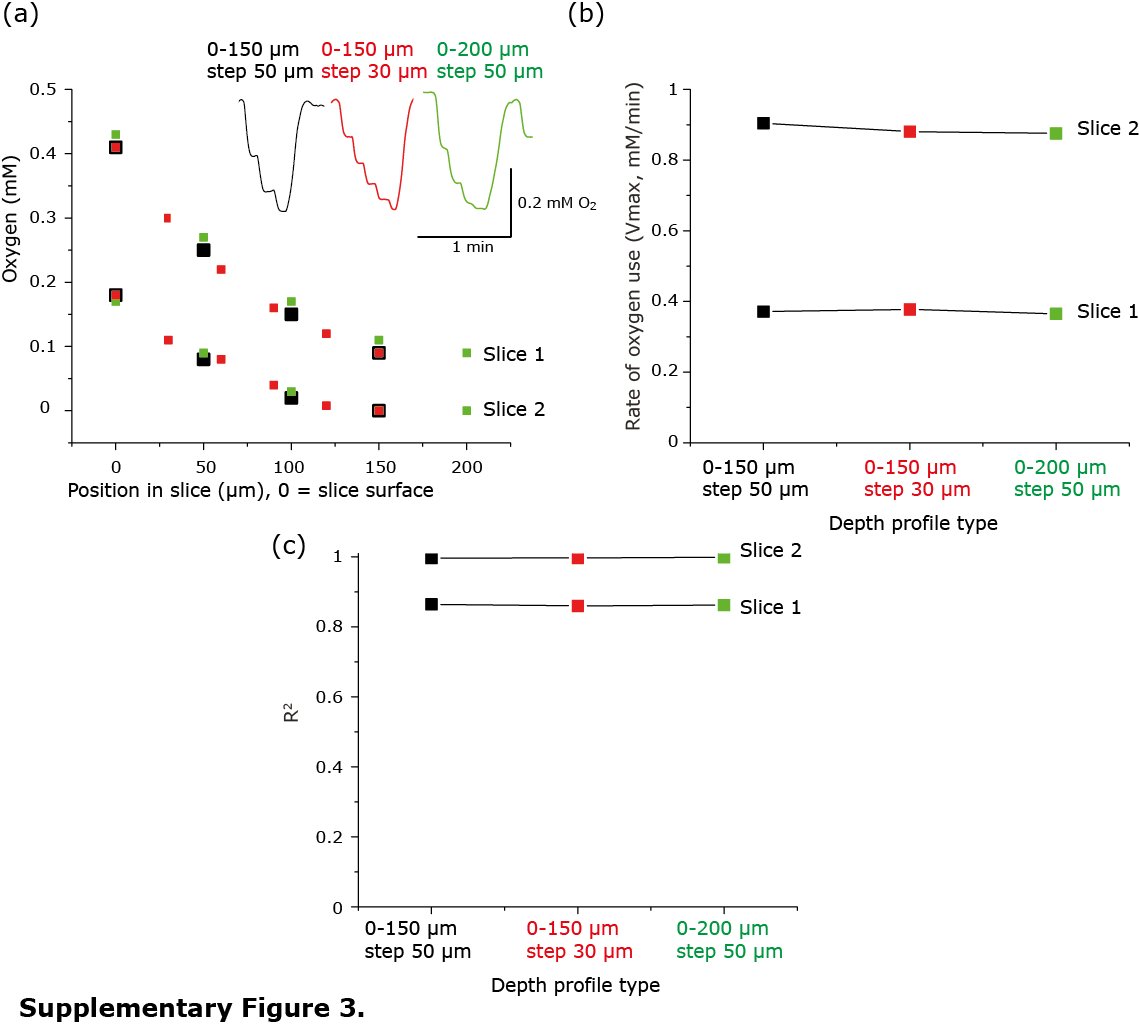


**Supplementary Figure 3.**

Different ways of obtaining the depth profile of [O_2_] do not affect the oxygen concentration profile through the slice (a), the calculation of oxygen consumption in the slice (b), or the goodness of fit of the theoretical equation to the data (c). (a) Oxygen level measurements through two sample slices using 3 types of spatial sampling: (1) from 0 to 150 µm in the slice, in steps of 50 µm (black), which is the depth sampling used throughout this paper, (2) from 0 to 150 µm in the slice, in steps of 30 µm (red), and (3) from 0 to 200 µm in the slice, in steps of 50 µm (green). (b) V_max_, the maximum rate of oxygen use, was obtained by fitting the three types of depth profile as described in the Materials and Methods. (c) The proportion of the sum of the squared residuals explained by the fit (R^2^) was not affected by the type of depth profile used.

**Supplementary References**

1 Shao L-R, Dudek FE. Increased excitatory synaptic activity and local connectivity of hippocampal ca1 pyramidal cells in rats with kainate-induced epilepsy. *J Neurophysiol* 2004; 92: 1366–1373.

2 Staff NP, Jung H-Y, Thiagarajan T, Yao M, Spruston N. Resting and active properties of pyramidal neurons in subiculum and CA1 of rat hippocampus. *J Neurophysiol* 2000; 84: 2398–2408.

3 Groc L, Gustafsson B, Hanse E. Spontaneous unitary synaptic activity in CA1 pyramidal neurons during early postnatal development: constant contribution of AMPA and NMDA receptors. *J Neurosci* 2002; 22: 5552–5562.
